# Characteristics and Outcome of *Streptococcus pneumoniae* Endocarditis in the XXI Century: A Systematic Review of 111 Cases (2000–2013)

**DOI:** 10.1097/MD.0000000000001562

**Published:** 2015-10-02

**Authors:** Viviana de Egea, Patricia Muñoz, Maricela Valerio, Arístides de Alarcón, José Antonio Lepe, José M. Miró, Juan Gálvez-Acebal, Pablo García-Pavía, Enrique Navas, Miguel Angel Goenaga, María Carmen Fariñas, Elisa García Vázquez, Mercedes Marín, Emilio Bouza, the GAMES Study Group

**Affiliations:** From the Microbiology and Infectious Diseases Department, Hospital General Universitario Gregorio Marañón (VDE, PM, MV, MM, EB); Department of Medicine, Universidad Complutense, Madrid (UCM), Spain (PM, MM, EB); CIBER de Enfermedades Respiratorias, Instituto de Salud Carlos III (CIBERES), Madrid, Spain (PM, MM, EB); Instituto de Investigación Sanitaria Gregorio Marañón (IiSGM) Madrid, Spain (PM, MV, MM, EB); Microbiology and Infectious disease department, Hospital Universitario Virgen del Rocio, Sevilla (ADA, JAL); Infectious Diseases Department. Hospital Clinic-IDIBAPS, University of Barcelona (Barcelona) (JMM); Department of Medicine, Unidad Clínica Intercentros de Enfermedades Infecciosas, Microbiolo gía y Medicina Preventiva Hospital Universitario Virgen Macarena. Departamento de Medicina. Universidad de Sevilla (JG-A); Department of Cardiology, Hospital Universitario Puerta de Hierro Majadahonda, Madrid (PG-P); Hospital Ramón y Cajal, Madrid (EN); UEI HU Donostia, San Sebastián (MAG); Hospital Marqués de Valdecilla, University of Catabria, Santander (MCF); and Hospital Clínico Universitario Virgen de la Arrixaca, Instituto Murciano de Investigación Biosanitaria (IMIB), Facultad de Medicina-Universidad de Murcia, Spain (EGV).

## Abstract

*Streptococcus pneumoniae* is an infrequent cause of severe infectious endocarditis (IE). The aim of our study was to describe the epidemiology, clinical and microbiological characteristics, and outcome of a series of cases of *S. pneumoniae* IE diagnosed in Spain and in a series of cases published since 2000 in the medical literature.

We prospectively collected all cases of IE diagnosed in a multicenter cohort of patients from 27 Spanish hospitals (n = 2539). We also performed a systematic review of the literature since 2000 and retrieved all cases with complete clinical data using a pre-established protocol. Predictors of mortality were identified using a logistic regression model.

We collected 111 cases of pneumococcal IE: 24 patients from the Spanish cohort and 87 cases from the literature review. Median age was 51 years, and 23 patients (20.7%) were under 15 years. Men accounted for 64% of patients, and infection was community-acquired in 96.4% of cases. The most important underlying conditions were liver disease (27.9%) and immunosuppression (10.8%). A predisposing heart condition was present in only 18 patients (16.2%). Pneumococcal IE affected a native valve in 93.7% of patients. Left-sided endocarditis predominated (aortic valve 53.2% and mitral valve 40.5%). The microbiological diagnosis was obtained from blood cultures in 84.7% of cases. In the Spanish cohort, nonsusceptibility to penicillin was detected in 4.2%. The most common clinical manifestations included fever (71.2%), a new heart murmur (55%), pneumonia (45.9%), meningitis (40.5%), and Austrian syndrome (26.1%). Cardiac surgery was performed in 47.7% of patients. The in-hospital mortality rate was 20.7%. The multivariate analysis revealed the independent risk factors for mortality to be meningitis (OR, 4.3; 95% CI, 1.4–12.9; *P* < 0.01). Valve surgery was protective (OR, 0.1; 95% CI, 0.04–0.4; *P* < 0.01).

*Streptococcus pneumoniae* IE is a community-acquired disease that mainly affects native aortic valves. Half of the cases in the present study had concomitant pneumonia, and a considerable number developed meningitis. Mortality was high, mainly in patients with central nervous system (CNS) involvement. Surgery was protective.

## INTRODUCTION

Invasive pneumococcal disease (IPD) remains a major health problem that affects 20 to 35,000 patients per year in the USA and Europe and causes 3500 to 5800 related deaths.^1,2^

*Streptococcus pneumoniae* was responsible for 15% of all cases of IE in the preantibiotic era,^3,4^ whereas in the 1980 to 1990s prevalence was <3%.^3,5^ However, recent data on the incidence of pneumococcal IE (PIE) are lacking.

Diagnosis, treatment, and outcome have improved during the last 15 years, thanks to routine immunization, new rapid molecular and imaging techniques, new cutoff minimum inhibitory concentration (MIC) criteria for penicillin sensitivity, and multidisciplinary management^6^.

Most major studies on PIE were published before the year 2000.The objectives of this study were to analyze the epidemiology and characteristics of PIE in a large prospective multicenter series and to review cases of PIE reported during the last 14 years.

## MATERIAL AND METHODS

### Setting and Study Design

We used the database of GAMES (*Grupo de Apoyo al Manejo de la EndocarditiS*), which is a prospective Spanish registry of consecutive patients with IE defined according to the modified Duke criteria^7^ diagnosed between January 1, 2008 and December 31, 2013 in 27 Spanish hospitals (n = 2539). Multidisciplinary teams completed a standardized case report form. Regional and local (Comité Ético de Investigación Clínica Hospital General Universitario Gregorio Marañón - Área 1) ethics committees approved the study, and patients gave their informed consent. The follow-up period lasted 1 year.

In order to increase the number of cases, we also included 4 patients prospectively recruited in the coordinating center from 2004 to 2008 (n = 228). Additionally, the literature was reviewed for articles in English, French, and Spanish published from 2000 to 2013 using the medical subject headings “*Streptococcus pneumoniae* endocarditis”, “pneumococcal endocarditis”, and “pneumococcus endocarditis”. We also searched reference lists to identify additional reports of *S. pneumoniae* endocarditis. If necessary, the authors were contacted in order to obtain additional information. Cases with insufficient clinical information were excluded from this analysis. All cases recorded during the study period (2000–2013) were included in a database for statistical analysis.

Diagnosis of IE was based on the Duke criteria combined with identification of *S. pneumoniae* in blood and/or in valve tissue. Identification was based on traditional microbiologic cultures or molecular techniques.

The IE episode was considered community-acquired or health care associated based on the classification of the International Collaboration on Endocarditis study group (ICE).^8^ Predisposing conditions for IE were registered, including previous valve disease, previous valve replacement, and presence of intracavitary devices, including pacemakers and implantable cardioverter defibrillators. Mortality during hospitalization and mortality after follow-up was recorded. The new values introduced in 2008 by the Clinical & Laboratory Standards Institute (CLSI) were used to determine susceptibility to penicillin and cefotaxime in the Spanish cohort.^9^ In the cases from the literature, when MIC values were not provided, the published susceptibility (resistant or susceptible) was accepted.

### Statistical Analysis

We calculated the incidence of *S. pneumoniae* endocarditis as the number of episodes detected each year divided by the number of inhabitants in the hospital catchment area (in hundreds of thousands) and by the number of hospital admissions (in thousands).

The statistical analysis was carried out using SPSS 15.0 (SPSS, Chicago, IL). In the univariate analysis, categorical variables were compared using the chi-square test or the Fisher's exact test. Non-normally distributed continuous variables were compared using the *t* test, and normally distributed variables were compared using the *t* test or analysis of variance. In order to assess potential changes in the characteristics of pneumococcal endocarditis, we compared cases from the Spanish cohort with cases collected from the literature review. Independent factors related to in-hospital mortality were also assessed using a backwards logistic regression model adjusted for sex, meningitis (yes vs no), and surgical treatment for the endocarditis episode (yes vs no).

## RESULTS

### Epidemiology

We analyzed 111 patients with PIE: 24 cases from the Spanish GAMES cohort (Table 1 ) and 87 from the literature published between 2000 and 2013.^4,10–70^ We excluded cases diagnosed before 2000^6,22,26,62,71,72^ and cases for which clinical information was insufficient.^73–79^

**TABLE 1:**
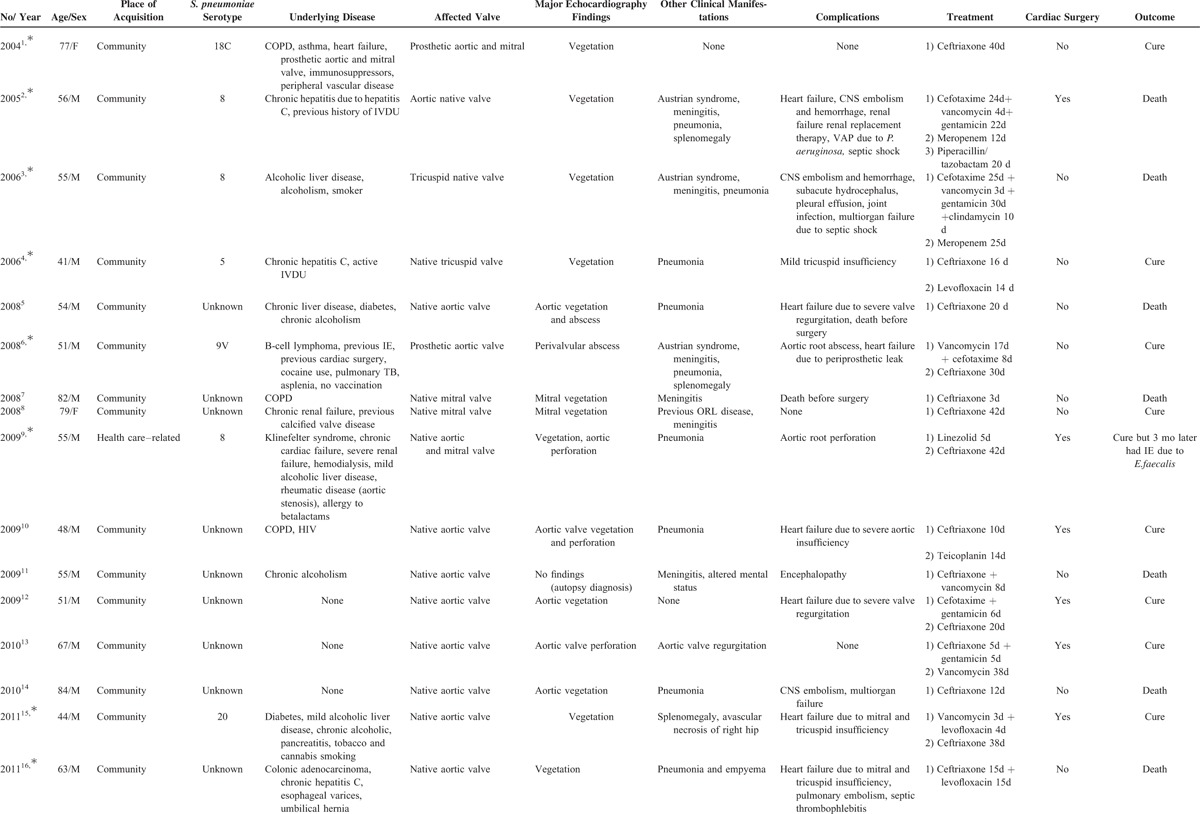
Characteristics of 24 Patients With *Streptococcus pneumoniae* Infective Endocarditis From the GAMES Cohort (2004–2013)

**TABLE 1 (Continued):**
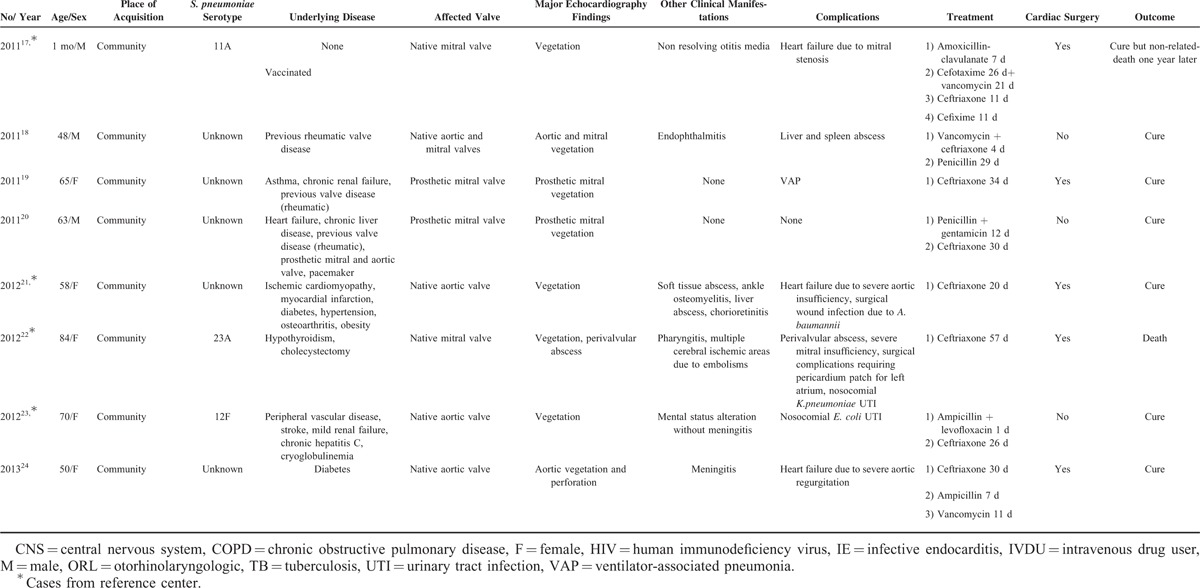
Characteristics of 24 Patients With *Streptococcus pneumoniae* Infective Endocarditis From the GAMES Cohort (2004–2013)

*Streptococcus pneumoniae* was identified in 24 cases, namely, 0.86% of the 2539 IE episodes registered in the GAMES cohort.

### Epidemiological Characteristics

The epidemiological characteristics of the 111 patients and the comparison between the Spanish series and the cases from the literature are shown in Table 2. Median age was 51 years (IQR, 26–63) and 71 patients were men (64%). All but 4 episodes (3.6%) were community acquired. The most common underlying condition was liver disease (27.9%), followed by immunosuppression (10.8%) and splenectomy or asplenia (8.1%). A predisposing heart condition was present in 20 patients (18%): 11 with congenital heart disease, 8 with previous valve disease (2 prosthetic valves), and 1 patient with a previous history of IE and valve replacement. Patients from the Spanish cohort were significantly older than those from the literature, with a median age of 57 (50–69) versus 47 (15–61) years (*P* 0.001), and more frequently had previous liver disease (45.8% vs 23% *P* 0.03) and predisposing heart valve conditions (27.3% vs 2.4% *P* 0.001).

**TABLE 2:**
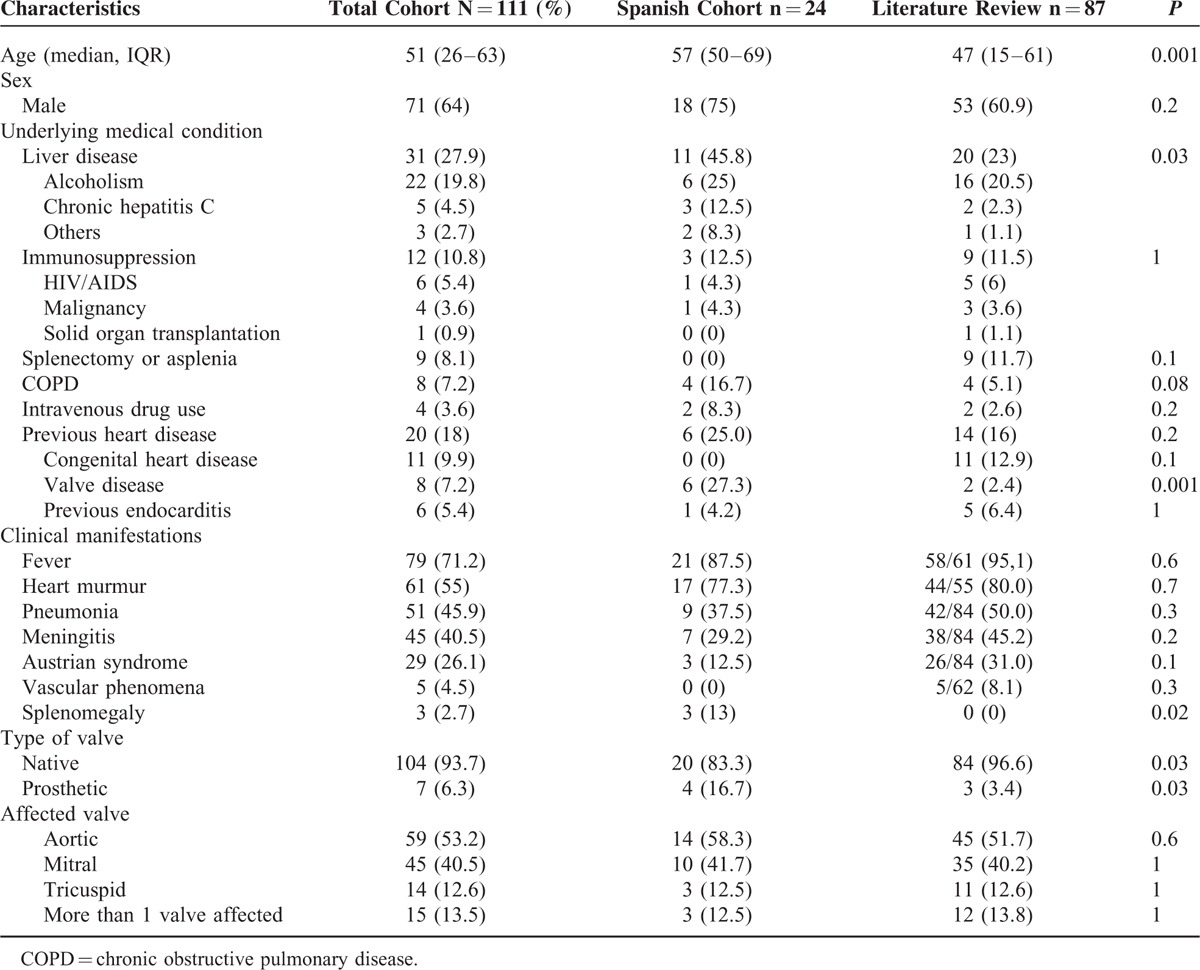
Demographic and Clinical Characteristics of 111 Patients With *Streptococcus pneumoniae* Endocarditis

### Clinical Presentation

Considering all cases, the most prevalent clinical manifestations were fever (71.2%), new heart murmurs (55%), and vascular phenomena that were only present in 5 patients (4.5%): 2 with disseminated petechiae, 1 with Osler nodes and Janeway lesions, 1 with subconjunctival hemorrhage and Roth spots, and 1 case with splinter hemorrhages. At diagnosis of endocarditis, 42% of patients presented concomitant pneumonia and 40.5% presented meningitis. Complete Austrian syndrome (endocarditis, meningitis, and pneumonia) was present in 29 patients (26.1%) (Table 2). PIE was predominantly left-sided (aortic valve in 53.2% and mitral valve in 40.5%) and involved a native valve in 93.7% of cases.

The Spanish cohort more commonly presented prosthetic valve endocarditis than the cases collected from the literature (16.7% vs 3.4%, *P* 0.03).

### Diagnostic Methods

Considering the whole series*, S. pneumoniae* was recovered from blood cultures in 84.7% of the patients and from other cultures in 73.3% of the cases where data were available. Cerebrospinal fluid was positive in 33.3% and valve cultures in 21.2% (Table 3).

**TABLE 3:**
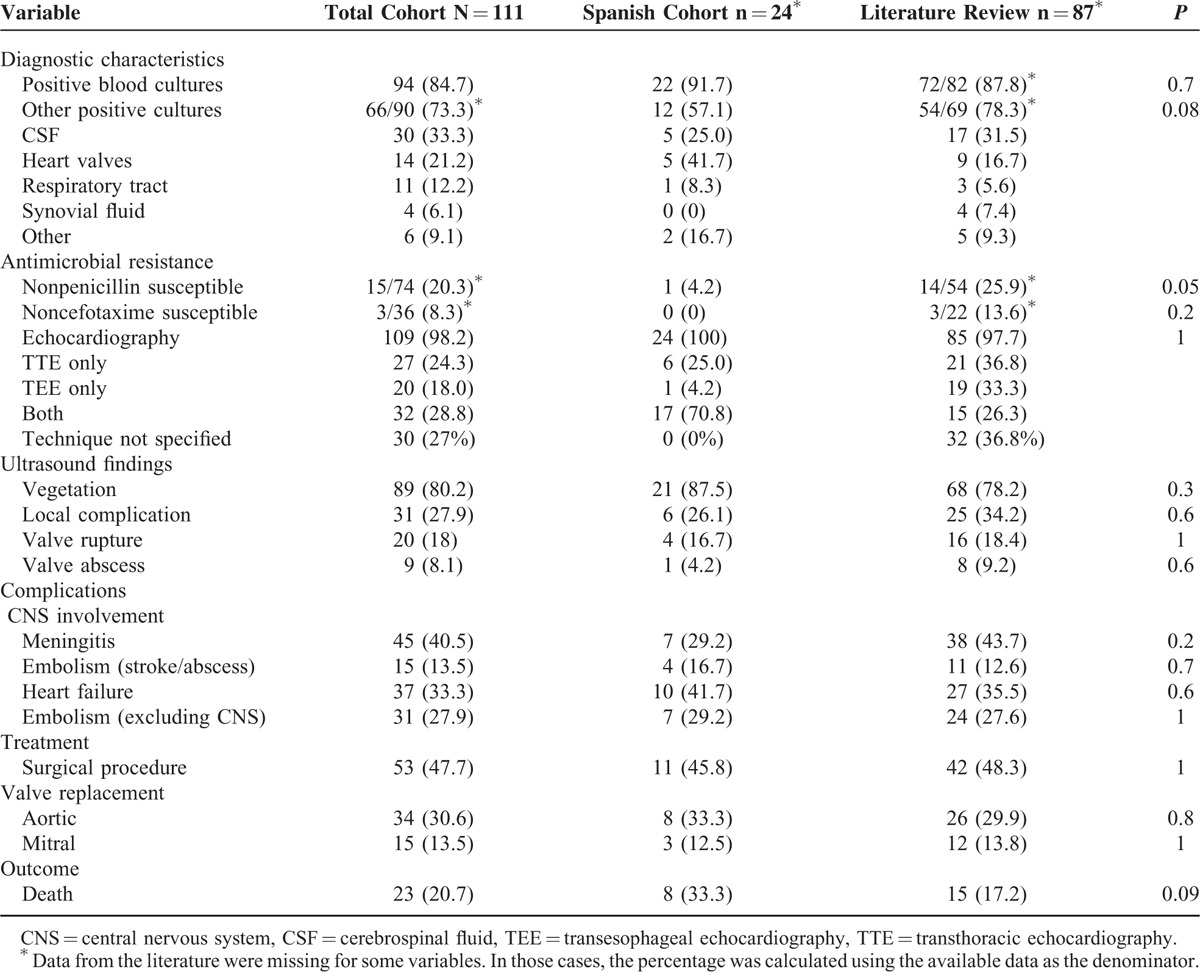
Diagnostic Characteristics and Complications of 111 Patients With Pneumococal Endocarditis

Data regarding molecular techniques performed on valve tissue were available for 9 patients of the 24 cases from the Spanish cohort; the results were positive for *S. pneumoniae* for all of them.

Furthermore, in the Spanish cases, antimicrobial susceptibility was analyzed using the new cutoffs introduced in 2008, whereas in most reports from the literature, MIC values were not included. Taking this characteristic limitation of retrospective data into account, penicillin nonsusceptibility rates were 4.2% in the Spanish cohort and 25.9% in the literature (*P* = 0.05).

Data on capsular serotypes were available for 27 cases from the whole series and revealed marked diversity. The most common serotypes were 18C, 23F, and 8 (3 each). Vaccination data were available for only 13 patients, of whom 4 had been vaccinated. We could not demonstrate any association between the serotype and clinical manifestations, complications, or patient outcome.

For the entire cohort, echocardiography was performed in 98.2% of patients (Table 3). Both of the 2 patients without echocardiographic data died before diagnosis, which was confirmed by autopsy. Major findings included vegetations (80.2%), valve rupture (18%), and abscesses (8.1%).

### Treatment and Outcome

Analyses of the whole series revealed that third-generation cephalosporins were used in 90.1% of the patients, vancomycin in 44.1%, and penicillin in 16.2%. Combined therapy was administered in 62.5% of cases and the most common combination was third-generation cephalosporins with vancomycin (15%). Almost half of the patients underwent cardiac surgery for the IE episode (47.7%) (Table 3). There was an indication for surgery in 67.4% of the patients, and the indications were: inadequate source of infection control or severe sepsis (17.7%), heart failure (12.5%), myocardial extension of the infection (ring abscess, perivalvular tissue involvement, or aortic fistula) (8.3%), valve rupture or severe valve malfunction (6.3%), systemic and CNS embolism (3.1%), IE after prosthetic valve replacement (2%). More than 1 indication was present in 11.5% of the cases. All of these events represent complications of the IE episode. Regarding the time of the surgery, 50% of patients underwent surgery <1 week after the IE diagnostic. We could not found any differences in the outcome of the patients regarding time of surgery.

The most common complications were heart failure (33.3%) and central nervous system embolisms (13.5%). Non-CNS embolism was reported in 31 patients (27.9%), including septic arthritis (7.2%) and endophthalmitis (6.3%).

Related mortality was 20.7%. As expected, mortality was higher in the Spanish cohort (33% vs 17%, *P* = 0.09), which included patients with more severe conditions (older age, more underlying diseases, and more frequent prosthetic valve involvement). The risk factors for mortality identified in the univariate analysis were peripheral embolism (47.8% vs 22.7%, *P* = 0.03), CNS embolism (34.8% vs 8%, *P* = 0.003), meningitis (65.2% vs 34.1%, *P* = 0.009), and Austrian syndrome (43.5% vs 21.6%, *P* = 0.059), and are summarized in Table 4. Cardiac surgery was a protective factor against mortality (17.4% vs 55.7%, *P* < 0.01). The multivariate analysis demonstrated that the independent risk factors for mortality were meningitis (OR, 4.3; 95% CI, 1.4–12.9 *P* < 0.01), whereas valve surgery was protective (OR, 0.1; 95% CI, 0.04–0.4; *P* < 0.01).

**TABLE 4:**
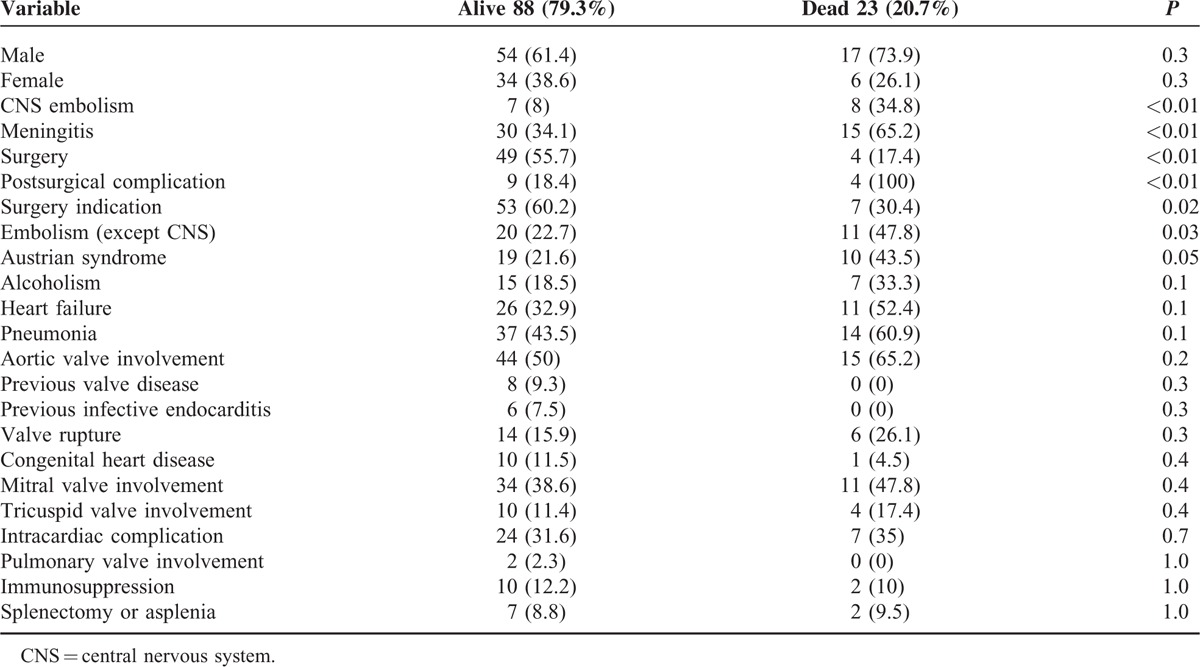
Prognostic Factors for 111 Patients With *Streptococcus pneumoniae* IE (Univariate Analysis)

## DISCUSSION

We performed a 14-year retrospective study of the epidemiology, clinical manifestations, complications, and outcome of PIE. PIE affected young adult patients with previous liver disease and alcoholism, even those with no history of valve disease. It is more frequent in left-sided valves. Interestingly, pneumonia was present in half of all cases and Austrian syndrome in a quarter. Despite advances in modern medicine, mortality remains high (20.7%), and complications due to valve dysfunction are common. Almost half of the patients in our study required valve surgery, and the only risk factor associated with better survival was early heart surgery.

In a publication of the International Collaboration on Endocarditis-Prospective Cohort, the prevalence of all types of streptococcal IE was 6% (excluding *viridans* group streptococci and *Streptococcus bovis*).^80^ No recent prospective data are available for PIE. The US Invasive Pneumococcal Disease (IPD) surveillance report showed 33,500 cases of IPD (10.6/100,000) and 3500 deaths (1.1/100,000).^1^ In Europe, according to the European Centre for Disease Prevention and Control, 26 EU/EEA countries reported 20,843 confirmed cases of IPD in 2011 (5.59/100,000), with 5771 related deaths (10.3%).^2^

Immunosuppressed patients (solid organ transplant, malignancy, and HIV infection) and patients with specific chronic disorders (cirrhosis, alcoholism, COPD, and diabetes mellitus) have a higher risk of developing IPD. Moreover, several studies proposed that these factors are even more important than the specific pneumococcal serotype as determinants of outcome.^3,81,82^ In the present series, the most common underlying conditions were liver disease due to alcoholism (19.8%) and immunosuppression (10.8%). Alcoholism is a well-known predisposing factor for IPD, and its frequency varies from 10.4% to 28.1%.^3,83^ The reasons for this observation are far from clear, although potential explanations include malnutrition, immunologic defects such as impaired leukocyte chemotaxis, reticuloendothelial system dysfunction, and risk of lung aspiration.^3,71^ Immunosuppression affected 10.8% of the patients in our study, mainly those with HIV infection (5.4%) and malignancy (3.6%). Interestingly, a previous study of IPD in our institution reported a higher percentage of immunosuppression (44%), of which the most common causes were HIV infection (13%) and malignancy (31%).^83^ These differences could be explained by the availability of highly active antiretroviral therapy and good immunological control of HIV-infected patients in recent years. Finally, previous liver disease was reported in 27.9% of cases; other authors reported previous liver disease in 12% of cases.^6,83^

The classic clinical manifestations of PIE include fever and acute pneumonia with progressive heart failure, which may be fatal if appropriate treatment is not administered early.^4,74^ We found pneumonia to be the first clinical manifestation of IPD in almost half of our cases (45.9%). The classic Austrian syndrome is characterized by meningitis, pneumonia, and endocarditis due to *S. pneumoniae* and is commonly associated with alcoholism. It was present in 26.1% of our series, and the frequency reported in the literature varied from 6.7% to 42% of cases.^3,71^ Austrian syndrome seems to be a poor prognostic factor and was present in 43.5% of those who died in our series.

PIE mainly affects native valves, as observed in 93.7% of the cases we report and 87 to 92% of those reported elsewhere.^6,71^ Left-sided endocarditis is the most common presentation, although data on the most commonly affected valves are contradictory and vary between series. Aortic valve involvement ranges from 46.7% to 74.4%, and mitral valve involvement ranges from 31.4% to 56.7%. We found that the aortic valve was the most commonly involved (53.2%).

Literature from the early 20th century suggested that PIE tends to form large vegetations that predispose to systemic embolism.^84,85^ In our study, almost one-third of patients developed systemic embolism (13.5% involving the CNS), and meningitis was present in 40.5%.

Starting in the 1990s, the prevalence of *S. pneumoniae* strains with decreased susceptibility to penicillin increased worldwide. In Spain, it ranged from 18.3% to 59%.^86,87^ However, resistance to penicillin has been decreasing in recent years, possibly owing to the change in the definition of the breakpoint penicillin MIC in 2008.^9^ A recent Spanish surveillance study found 28% prevalence of penicillin-resistant strains when the CNS infection cutoff MIC of >0.06 mg/L was applied; however, when the cutoff for non-CNS infection was applied (MIC >2 mg/L), the isolates all proved to be susceptible to penicillin.

According to European guidelines, CNS involvement (meningitis) requires treatment with ceftriaxone or cefotaxime either alone or in combination with vancomycin.^8^ In the present review, we found that 20.3% of strains were classified as nonsusceptible, although the specific MIC values were not available in the cases from the literature. In contrast, in the Spanish cohort, where MIC values were available, we calculated that with the new cutoffs, nonpenicillin-susceptible strains only accounted for 4.2%. Nevertheless, we were not able to find an association between susceptibility to penicillin and clinical outcome.

Information on capsular serotypes was scarce in the literature and in our series; the most common serotypes were 18C, 23F, and 8. The correlation between pneumococcal serotypes and outcome is controversial. Some studies highlight the relationship between *S. pneumoniae* serotypes and the risk of septic shock and higher mortality,^82,88^ whereas others do not and conclude that host factors are better determinants of clinical outcome. The above-mentioned lack of data on capsular serotype from many of the patients included in this series represents a limitation for the analysis of its impact. Considering this, we could not demonstrate any association between capsular serotype and clinical manifestations, complications, or patient outcome.

As for the epidemiological impact of the pneumococcal vaccines, numerous studies show a marked decrease in the prevalence of IPD.^89–93^ In our series, it was difficult to prove a correlation between vaccination status and severity of infection. In Spain, data that would enable us to resolve this question are lacking. A systematic vaccination register is recommendable to evaluate whether vaccination has had an impact in the incidence of IPD.

Transesophageal echocardiography was performed in 75% of cases in the Spanish cohort and in 61.8% of cases from the literature. The major echocardiographic findings were vegetations in 80.2% of patients and local complications such as valve rupture and perivalvular abscess in 26.1%.

Almost half of the cohort underwent valve replacement for the IE episode. The most frequent indications for surgery in this series were inadequate control of infection source, severe sepsis, heart failure, and myocardial extension of the infection. In the multivariate analysis, this procedure was the only protective factor against mortality. In the ICE multicenter study, where ∼50% of patients underwent surgery, it was also reported that early surgery improved clinical outcome. Global mortality for the ICE cohort was 18%.^80^ Other series presented PIE-related mortality rates ranging between 24.1% and 62%.^3,6,71^

In summary, PIE continues to be a severe but very infrequent community-acquired infection. It affects patients with predisposing underlying conditions and can cause complications, such as meningitis and embolism and severe heart failure. The new MIC breakpoints for penicillin make high-dose penicillin or a third-generation cephalosporin the drugs of choice. Mortality remains high despite early diagnosis and treatment, which is frequently carried out by multidisciplinary groups. Early surgery should always be considered.

## APPENDIX 1

**Members of GAMES: Hospital Costa del Sol**, (Marbella): Fernando Fernández Sánchez, Marian Noureddine, Gabriel Rosas, Javier de la Torre Lima; **Hospital Universitario de Cruces**, (Bilbao): José Aramendi, Elena Bereciartua, María Victoria Boado, Marta Campaña Lázaro, Josune Goikoetxea, Juan José Goiti, José Luis Hernández, José Ramón Iruretagoyena, JosuIrurzun Zuazabal, Leire López-Soria, Miguel Montejo, Pedro María Pérez, Regino Rodríguez, Roberto Voces; **Hospital Universitario Virgen de la Victoria**, (Málaga): Mª Victoria García López, Radka Ivanova Georgieva, Manuel Márquez Solero, Isabel Rodríguez Bailón, Josefa Ruiz Morales; **Hospital Universitario Donostia-Policlínica Gipuzkoa**, (San Sebastián): Ana María Cuende, Tomás Echeverría, Ana Fuerte, Eduardo Gaminde, Miguel Ángel Goenaga, Pedro Idígoras, José Antonio Iribarren, Alberto Izaguirre Yarza, Carlos Reviejo; **Hospital General Universitario de Alicante**, (Alicante): Rafael Carrasco, Vicente Climent, Patricio Llamas, Esperanza Merino, Joaquín Plazas, Sergio Reus; **Complejo Hospitalario Universitario A Coruña Hospital Juan Canalejo**, (A Coruña): Nemesio Álvarez, José María Bravo-Ferrer, María del Mar Carmona, Laura Castelo, José Cuenca, Pedro Llinares, Enrique Miguez Rey, María Rodríguez Mayo, Dolores Sousa, Mª Carmen Zúñiga; **Complejo Hospitalario de Especialidades Juan Ramón Jiménez**, (Huelva): Francisco Javier Martínez; **Hospital Universitario de Canarias**, (Canarias): Mª del Mar Alonso, Beatriz Castro, Dácil García Marrero, Mª del Carmen Durán, Mª Antonia Miguel Gómez, Juan La Calzada, Ibrahim Nassar; **Hospital Regional Universitario Carlos Haya**, (Málaga): Antonio Plata Ciezar, José Mª Reguera Iglesias; **Hospital Universitario Central Asturias**, (Oviedo): Víctor Asensi Álvarez, Carlos Costas, Jesús de la Hera, Jonnathan Fernández Suárez, José Manuel García Ruiz, Lisardo Iglesias Fraile, José López Menéndez, Pilar Mencia Bajo, Carlos Morales, Alfonso Moreno Torrico, Carmen Palomo, Begoña Paya Martínez, Ángeles Rodríguez, Raquel Rodríguez García, Mauricio Telenti; Hospital Clinic-IDIBAPS, University of Barcelona (Barcelona): Manuel Almela, Yolanda Armero, Manuel Azqueta, Mercé Brunet, Ramón Cartañá, Carlos Cervera, Carlos Falces, Guillermina Fita, David Fuster, Cristina García de la Maria, José M. Gatell, Jaume Llopis Pérez, Francesc Marco, Carlos A. Mestres, José Mª Miró, Asunción Moreno, Salvador Ninot, Eduardo Quintana, Carlos Paré, Juan Manuel Pericás, José L. Pomar, José Ramírez, Irene Rovira, Marta Sitges, Dolors Soy, Adrián Téllez, Jordi Vila; **Hospital General Universitario Gregorio Marañón**, (Madrid): Javier Bermejo, Emilio Bouza, Viviana de Egea, Alia Eworo, Ana Fernández Cruz, Mª Eugenia García Leoni, Marcela González del Vecchio, Víctor González Ramallo, Martha Kestler Hernández, Mercedes Marín, Manuel Martínez-Sellés, Mª Cruz Menárguez, Patricia Muñoz, Cristina Rincón Hugo Rodríguez-Abella, Marta Rodríguez-Créixems, Jorge Rodríguez Roda, Blanca Pinilla, Ángel Pinto, Maricela Valerio, Eduardo Verde Moreno; **Hospital Universitario La Paz**, (Madrid): Isabel Antorrena, Mar Moreno, José Ramón Paño, Sandra Rosillo, María Romero, Araceli Saldaña; **Hospital Universitario Marqués de Valdecilla**, (Santander): Carlos Armiñanzas Castillo, Ana Arnaiz, José de Berrazueta, Manuel Cobo Belaustegui, Raquel Durán, Mª Carmen Fariñas, Concepción Fariñas-Álvarez, Carlos Fernández Mazarrasa, Rubén Gómez Izquierdo, Claudia González Rico, Manuel Gutiérrez-Cuadra José Gutiérrez Díez, Rafael Martín Durán, Marcos Pajarón, José Antonio Parra, Ramón Teira, Jesús Zarauza; **Hospital Universitario Puerta de Hierro**, (Madrid): Pablo García Pavía, Jesús González, Beatriz Orden, Antonio Ramos, Elena Rodríguez González; **Hospital Universitario Ramón y Cajal**, (Madrid): Tomasa Centella, José Manuel Hermida, José Luis Moya, Pilar Martín-Dávila, Enrique Navas, Enrique Oliva, Alejandro del Río, Soledad Ruiz; **Hospital Universitario Virgen de las Nieves**, (Granada): Carmen Hidalgo Tenorio; **Hospital Universitario Virgen Macarena**, (Sevilla): Antonio de Castro, Marina de Cueto, Pastora Gallego, Juan Gálvez Acebal, Jesús Rodríguez Baño; **Hospital Universitario Virgen del Rocío**, (Sevilla): Arístides de Alarcón, Emilio García, Juan Luis Haro, José Antonio Lepe, Francisco López, Rafael Luque; **Hospital San Pedro**, (Logroño): Luis Javier Alonso, José Manuel Azcona Gutiérrez, José Ramón Blanco, Lara García, José Antonio Oteo; **Hospital de la Santa Creu i Sant Pau**, (Barcelona): Natividad de Benito, Mercé Gurguí, Cristina Pacho, Roser Pericas, Guillem Pons; **Complejo Hospitalario Universitario de Santiago de Compostela**, (A Coruña): M. Álvarez, A. L. Fernández, Amparo Martínez, A. Prieto, Benito Regueiro, E. Tijeira, Marino Vega; **Hospital Santiago Apóstol**, (Vitoria): Andrés Canut Blasco, José Cordo Mollar, Juan Carlos Gainzarain Arana, Oscar García Uriarte, Alejandro Martín López, Zuriñe Ortiz de Zárate, José Antonio Urturi Matos; **Hospital SAS Línea de la Concepción**, (Cádiz): Mª Belén Nacle, Antonio Sánchez, Luis Vallejo; **Hospital Universitario Virgen de la Arrixaca** (Murcia): José Mª Arribas Leal, Elisa García Vázquez, Alicia Hernández Torres, Ana Blázquez, Gonzalo de la Morena Valenzuela; **Hospital de Txagorritxu**, (Vitoria): Ángel Alonso, Javier Aramburu, Felicitas Elena Calvo, Anai Moreno Rodríguez, Paola Tarabini-Castellani; **Hospital Virgen de la Salud,** (Toledo): Eva Heredero Gálvez, Carolina Maicas Bellido, Mª Antonia Sepúlveda; **Hospital Rafael Méndez,** (Lorca-Murcia): Eva Cascales Alcolea, Pilar Egea Serrano, José Joaquín Hernández Roca.
